# The determinants of contraception use amongst female patients attending Odi District Hospital, Gauteng province, South Africa

**DOI:** 10.4102/safp.v62i1.5043

**Published:** 2020-08-25

**Authors:** Shango N. Olowa, Indiran Govender, Christian Saidiya

**Affiliations:** 1 Department of Family Medicine and Primary Health Care, Sefako Makgatho University, Pretoria, South Africa; 2 Department of Family Medicine and Primary Health Care, University of Pretoria, Pretoria, South Africa

**Keywords:** determinants, contraception, female patients, women, family planning, Odi District Hospital, South Africa

## Abstract

**Background:**

Empowering women to have a full control over the size of their family is not only an issue of human rights but also a sustainable development goal. This study sought to determine the factors influencing the use of contraception amongst female patients aged 18–49 years attending Odi District Hospital, Tshwane district.

**Methods:**

A cross-sectional survey was carried out from September 2018 to February 2019 at Odi District Hospital. A representative sample size of 400 female patients was recruited by systematic random sampling. Logistic regression model was used to determine the most influential predictors.

**Results:**

The mean age in the studied population was 30.65 (±7.57) years. Contraceptive prevalence was estimated to be 55.3%. Dual protection (condom) was used as additional method by up to 72.3% of respondents. Injectables remained the most used contraceptive method, while more permanent methods, such as Bilateral Tubal Ligation (BTL), were less utilised. The source of family planning information, past exposure to contraceptive methods and woman’s number of living children (parity) determined the use of contraception amongst Odi district females.

**Conclusion:**

This study revealed a discrepancy within the maternal health delivery system regarding the supply and demand chain prompting the need for more insights. The results suggest evidence-based reengineering programme that incorporates contraceptive uptake determinants into the maternal health delivery system.

## Background

The use of contraceptives is globally considered to be crucial towards fertility control.^1,2^ A high fertility rate, and hence high population growth, has been associated with an increased level of poverty and decreased life expectancy.^1,2,3^

It has been shown that the use of contraceptives significantly promotes maternal and child health not only by reducing the number of unplanned pregnancies but also by decreasing maternal deaths by up to 40%.^4^ Furthermore, the report released in 2015 by the United Nations confirmed that a reduction in total fertility rate from 4.7 births in the early 1970s to 2.6 births in women aged between 15 and 49 years was associated with a significant rise in worldwide use of contraceptives from 36% to 64%.^5^

Reproductive health programme offers a variety of family planning methods, and data from various sources show wide disparities between and within region and countries. In sub-Saharan Africa, the fertility rate remains higher, although the contraceptive use is substantially lower than anywhere else in developing countries.^5,6,7^ Women’s age, marital status, previous exposure to family planning, family planning information sources, geographical location, sexual behaviours, number of living children, culture, etc. have been presumed to be the determinants of contraception use.^6,8,9,10^

In South Africa, the determinants of contraception use are variable and multifactorial, with hormonal contraceptives (pills, injections) and sterilisation declining since 1998, although the use of male condoms for contraception was estimated at approximately 15% of the modern contraceptive methods used by women (58.3%).^11^ Although South Africa has made progress towards several Sustainable Development Goal (SDG) targets, progress report indicates that meeting the target towards SDG 5 (including sexual and maternal health) has been slow and wide variations still exist within and between provinces.^1,5,11^ The ideal contraception is a dual protection, which includes a barrier method (condom) to protect against sexually transmitted infections as well as pregnancy.^9,11^ This is especially appropriate in settings such as South Africa where the HIV and AIDS burden is high.^9,11^

Several international studies have been conducted in this regard, but few have been carried out in South Africa, particularly at Odi District Hospital where determinants of contraceptive use amongst female patients remain informal and unknown. Therefore, a cross-sectional survey conducted in this regard could provide valuable information.

Most studies in family planning usually look at the prevalence of contraceptives in a selected geographical location. These types of studies by themselves do not provide sufficient information to guide policy makers. Nevertheless, these studies coupled up with studies on the factors influencing the use of contraceptive methods, also called ‘determinants of contraceptive use’, could provide a comprehensive guide to policy makers and programme implementers in reproductive health. The present study sought to determine the factors that influence use of contraception amongst women in Odi district.

Contraception enables women to make informed choices about their sexual and reproductive health through the use of various devices, sexual practices, chemicals, drugs or surgical procedures.^12^ However, in the context of this study, the term ‘contraceptive’ refers to modern contraceptive methods such as pills, injectables, long-acting reversible contraceptives (e.g. intrauterine contraceptive device [IUCD] Implanon) and permanent methods (bilateral tubal ligation or BTL).

Women’s contraceptive behaviours have been studied extensively with or without a theoretical framework basis.^13^ However, in the context of the present research we chose to use a framework model that integrates intermediate determinants’ framework proposed by Davis and Blake (1956) and the fertility decision-making model presented by Bulatao and Lee (1983).^14,15^ The main concern is about finding the most influential factors that affect contraceptive outcomes.

Although it is almost impossible to include all variables and pathways in one model, the key purpose of the present study framework is to highlight how various components of decision-making process act as an intervening factor in influencing use of contraception.

The decision-making consists of three elements: knowledge, motivation (goals) and assessment of fertility regulation. The initial step involves *awareness* or *knowledge* of source of family planning information and other services, previous exposure to family planning and decisive age for the use of relevant method.

The second stage (motivation) involves couple’s or individual’s fertility preference or needs. This stage of decision-making process could be influenced by socio-economic, cultural and family life cycle patterns. The concept of *motivation or goals of contraception* use has been extensively studied in the economic models of fertility in which motivation is thus defined as the balance between supply and demand.^14^

The last stage in the decision-making process is assessment, which is the weighing of positives and negatives of adopting contraception method.^14,15^

The weighing is subject to competence, attitude and beliefs. *Contraceptive competence* is the ability of an individual woman or couple to use contraceptive methods; it comprises woman’s age, couple’s or woman’s level of education, occupation, etc.

*Attitudes* or *beliefs* about contraceptive use are considered to be of great importance regarding contraceptive intention or evaluation. Contraceptive evaluation is the assessment of ethical, moral and cultural influences affecting the use of contraceptives (e.g. religion, marital status, number of living children, race, etc.).^14,15^

The integration of two models leads to an assumption that woman’s decision to use contraceptives results from the alteration or selection of the most influential predictors within the presumed categorical variables (knowledge, motivation, competence and attitude or belief factors).

## Methods

### Study design

This was a cross-sectional survey conducted from 01 September 2018 to 28 February 2019.

### Study population

The study population comprised female patients who consented to participate in the study within the data collection period. These participants were residents of Mabopane, which is a township mostly occupied by black people (99.2%) as determined by 2011 census.^10^ The majority of residents understands and speaks both English and Tswana. Most of the residents are of Christian faith with different nominal sects such as Zion Christian Church, Lutheran, Twelve apostles and John Wesley. Non-Christian faith in the minority includes Islam.^10^

### Study sample

Systematic random sampling was used to recruit participants; every third eligible woman was approached to participate in the study over a period of 6 months (September 2018 to February 2019).

The number of women in childbearing age (15 to 49 years) in Mabopane (Odi district) according to the 2011 census report was estimated to be the two-thirds (37 373) of the total female (56 060) population of this region.^10^ However, because of the limitation of legal age for informed consent (Section 12(2)(b)) in the research, which is 18 years and older, females younger than 18 years were excluded from the study. Therefore, female patients aged 18 to 49 years were only included.

By using the below-mentioned sample size formula or the sample size calculator software (Raosoft version) with 95% confidence interval (CI), *Z*-score (the number of standard deviations a given proportion is away from the mean) of 1.96 and an error margin of 0.05, the statically significant sample size calculated from the census report (37 373) was 381.

Because all women who approached did not consent to participate, and to minimise sampling bias, we over-sampled by about 10%, resulting in a final sample size of 400 women.

$$\frac{\frac{z^{2}\times p(1-p)}{e^{2}}}{1+\left(\frac{z^{2}\times p(1-p)}{e^{2}N}\right)}$$ [Eqn 1]

where *N* is the population size, *e* is the error margin and *z* is the *Z*-score and *p* is the sample proportion.

### Study setting

The survey was conducted at Odi District Hospital located in Mabopane township, situated in the Tshwane Metropolitan Municipality, in the north of the Gauteng province.

The hospital is government-funded, 227-bedded institution, convenient for the local population and offering free healthcare services to all.^10^ It is at the same time a reference hospital for patients with a catchment area of over 42.20 km^2^. Furthermore, from the hospital data base or records, at least 300 patients per month were eligible for this study. It offers the following services: reproductive health; neonatal intensive care unit; surgery department; dental facilities; obstetrics facilities; trauma unit; eye department; and clinical laboratory service.^10,11^

### Data collection

Self-administered or assisted questionnaire was used for this study. The questionnaire was used in similar study and pre-tested; shortcomings such as ambiguity, irrelevancy and inconsistencies were removed to ensure validity and reliability.^16^

The participants were given a choice in regarding the language and their preferred method of administration of questionnaire: self-administered (alone) or assisted by the researcher and/or research assistant. The consent was obtained from each participant before the questionnaire was administered after careful explanation of the research project.

Participants were given adequate time to answer the questionnaire, which was available in both English and Setswana. A trained research assistant proficient in the local language (Setswana) assisted with the administration of questionnaire.

The research assistant had complete information regarding the purpose of the research, how to obtain informed consent and how to complete the questionnaire.

All patients who met the inclusion criteria and consented to participate in the study during the stipulated period were included in the study until the sample size was completed.

### Data analysis

The collected data were entered into a Microsoft Excel spreadsheet. As the response variable in the study had two categories (users and non-users), the binary logistic regression model was fitted to assess the net effect of selected socio-demographic variables. Differences in modern contraceptive use by socio-demographic characteristics were assessed by using chi-square analyses.

To find the determinants of contraceptive use, all variables significant in bivariate analyses were simultaneously entered into the stepwise logistic regression model, and the most influential factors were explored. Multi-colinearity in logistic regression analyses was checked by examining standard errors for regression coefficients.

Data were analysed by using SAS (SAS Institute Inc, Cary, NC, United States), release 9.4, running under Microsoft Windows for perosnal computers (PC).

### Ethical considerations

The study was commenced after the School Research Ethics Committee (SREC) and Sefako Makgatho University Research Ethics Committee (SMUREC) (SMUREC ethics reference number: SMUREC/M/212/2018: PG) approved the protocol. The research was also approved by Tshwane District Health Research committee (Project number: 77/2018) and Odi District Hospital Research Committee (Research project number: ODI-2018/10).

This study is also registered at the National Health Research database (NHDR), with reference number: GP-201809-016. Informed consent in English and Setswana was obtained from all the participants, and they were allowed to withdraw at any stage of the research.

## Results

The mean age (±standard deviation) of the studied population was 30.65 (±7.57). Out of 400 respondents, 221 (55.3%) were using modern contraceptives during the study period. Being an ideal contraceptive method according to the World Health Organization (WHO) and also the only method that could be used concomitantly with other methods, barrier method (condom) in this study was analysed as an additional method to other methods.

### Characteristics of respondents

Table 1 shows that injectable contraceptives were used by almost half (49.7%) of women, and choice of contraceptive method was significantly associated with condom use (*p* < 0.0001). Of the modern contraceptive methods, concomitant use of condom was adopted by 72.3% of females.

**TABLE 1:** Distribution of modern contraceptives as per choice of method.

Variables	Choice of method								Total		*p*
	Pills		Injectable		LARC		BTL				
	*n*	%	*n*	%	*n*	%	*n*	%	*n*	%	
**Additional condom use**											
Yes	33	14.9	86	38.9	19	8.6	22	9.9	160	72.3	< 0.0001
No	18	8.2	24	10.8	14	6.4	5	2.3	61	27.7	
**Total**	**51**	**23.1**	**110**	**49.7**	**33**	**15**	**27**	**12.2**	**221**	**100**	**-**

LARC, long-acting reversible contraceptive; BTL, bilateral tubal ligation.

Table 2 shows the distribution of contraceptive use as per the four components of our operational framework. At a bivariate level of analysis by using chi-square test, the result shows that women’s number of living children, their source of family planning information as well as previous exposure to contraceptives could be related to their current contraceptive practice. Contraceptive use was found to be the highest amongst female patients having one or two children (29.3%) whilst showing a relatively fair distribution amongst those who were previously exposed to contraceptives. The result also suggests that healthcare facilities (clinic/hospital) were the biggest reservoir of family planning information (users: up to 20.9% and non-users: up to 23.1%). The other factors have little or no influence on contraceptive use decision-making process (*p* > 0.05).

**TABLE 2:** Distribution of contraceptive use as per the four components of operational framework.

Predictor variables	Modern contraceptive use				*p* (Chi-square)
	Yes		No		
	*N*	%	*N*	%	
Frequency (%)	221	55.3	179	44.7	-
**Knowledge factors**					
**Sources of information**					0.01*
Friends or colleagues	54	13.7	37	9.4		
Parents or relatives	56	14.3	27	6.9	
Media	29	7.4	17	4.3	
Clinic or hospital	82	20.9	91	23.1	
**Onset age of use of contraception if ever used**					0.07
Less than 18 years	39	11.3	27	7.8	
18–25 years	148	42.9	73	21.1	
26–32 years	18	5.2	22	6.4	
33–40 years	10	2.9	6	1.7	
41–49 years	2	0.6	0	0.0	
**Previous exposure to family planning (duration in years) if ever used**					0.01*
Less than 1 year	57	16.5	51	14.8	
1–2 years	50	14.5	29	8.4	
3–4 years	58	16.8	19	5.5	
4 years or more	52	15.0	29	8.4	
**Motivation factors**					0.1
Demand for limiting	84	21.0	0	0.0	
Demand for pacing	103	25.7	0	0.0	
Other reasons of use	34	8.5	0	0.0	
Demand for conception	0	0.0	70	17.5	
Preferred method not available	0	0.0	8	2.0	
Fears of side effects	0	0.0	40	10.0	
Fears of infertility after use	0	0.0	31	7.7	
Other reasons	0	0.0	30	7.5	
**Competence factors**					
**Women’s age group (years)**					0.07
18–25	50	12.5	56	14	
26–33	91	22.7	53	13.3	
34–41	55	13.7	50	12.5	
42–49	25	6.3	20	5.0	
**Women employment**					0.07
Formally employed	92	23.0	57	14.3	
Unemployed	112	28.0	111	27.7	
Self-employed	17	4.3	11	2.7	
**Women’s education**					0.4
No education	4	1.0	6	1.5	
Primary	23	5.7	24	6.0	
Secondary	138	34.5	100	25	
Tertiary	58	14.0	49	12.3	
**Partner’s education**					0.2
Never went to school	11	3.0	5	1.4	
Primary	17	4.7	6	1.7	
Secondary	123	34.0	100	27.6	
Tertiary	54	14.9	46	12.7	
**Partner’s employment**					0.1
Employed	151	41.6	106	29.2	
Unemployed	54	14.9	52	14.3	
**Attitude or belief factors**					
**Marital status**					0.4
Singe	147	36.7	133	30.7	
Married	49	12.3	44	11.0	
Separated	14	3.5	8	2.0	
Divorce	8	2.0	2	0.5	
Widow	3	0.7	2	0.5	
**Religion**					0.3
Christian	186	46.5	156	39.0	
Muslim	13	3.3	12	3.0	
Other	22	5.5	11	2.7	
**Parity**					0.003*
No child	31	7.7	41	10.3	
1–2 children	117	29.3	103	25.7	
3 or more children	73 ()	18.3	35	8.7	
**Race**					0.2
Black	198	49.5	167	41.7	
Mixed race	18	4.5	7	1.7	
Others	5	1.3	5	1.3	

* , Significant results (*p* < 0.05).

### Determinants of contraception use amongst female patients

To identify the most influential predictors that might affect the likelihood of using contraceptives, all variables were included in the stepwise multinomial logistic regression model. The logistic regression analysis revealed that only three categorical variables shown in Table 3 were likely to influence the use of contraception in this community. The rest of variables were not significant statistically (*p* > 0.05).

**TABLE 3:** Logistic regression results for determinants of contraception use.

Predictors	Odds ratio	95% CI	*p*
**Parity**			0.05
No child	-	-	
1–2 children	0.379	0.136–1.057	
3 or more children†	0.434	0.215–0.876	
**Sources of information**			0.0007
Friends or colleagues	3.018	1.451–6.279	
Parents or relatives	3.111	1.538–6.293	
Media (TV, Internet, etc.)	3.716	1.493–9.250	
Clinic or hospital†	-	-	
**Previous exposure to Family Planning**			0.02
Less than 1 year	0.436	0.191–1.000	
1–2 years	0.736	0.325–1.668	
3–4 years	1.432	0.610–3.362		
4 or more years†	-	-	

CI, confidence interval.

† , Reference category.

Results in Table 3 show that the sources of family planning information and previous exposure to contraceptives were the most influential determinants (*p* < 0.05) in the studied population. The likelihood of using contraceptives increased (odds ratio, OR [95% CI]: 1.432 [0.610–3.362]) with the number of years previously spent with a contraceptive method. Women who had received family planning information from friends or colleagues, parents or relatives and media (TV, Internet etc.) were respectively 3.018, 3.111 and 3.716 times more likely to use contraceptives than their counterparts. Although variably predictive, woman’s number of living children (parity) could also be another determinant as the result suggested that the outcome could go either way (*p* = 0.05). The likelihood of using contraceptives increased (OR [95% CI]: 0.434 [0.215–0.876]) with an increase in the number of children.

## Discussion

This study assessed the relationship between socio-demographic factors and the use of contraception amongst women of reproductive age in Odi district (Mabopane).

Prevalence of contraception in the studied population was estimated at 55.3%. This trend was within the WHO global estimates (36% – 64%) and relatively similar to the 2017 South African statistics (58.3%) and the pattern observed during the national household survey on contraception coverage amongst women in South Africa (49.1%).^11,17,18^

The study also depicted injectables as the most dominant contraceptive method of choice (almost half of the users). The dominance of injectables was similarly reported in the various studies conducted in Tanzania (2014), United Arab Emirates (2016), South Africa (2017) and North-West Ethiopia (2018).^17,19,20,21^

Findings of the present study suggested that the most significant factors that could influence contraceptive uptake in Odi district were the sources of family planning information and the women’s past exposure to contraceptives.

The study demonstrated that women who received family planning education from a healthcare worker at a health establishment such as hospital or clinic were more likely to use contraceptives than their counterparts. This finding supported the WHO family planning policies and the South African standard treatment guidelines recommending that ‘the choice of contraception should be taken after consultation with a healthcare professional taking into account safety, efficacy, acceptability and access’.^18,22^ The source of information had also been studied in different studies and was found to influence contraceptive use.^20,23,24^

The study results also suggested that the likelihood of using contraceptives increased in women who were previously exposed to contraceptives for a much longer period than those exposed for a shorter period (less than a year). This finding was in support of the WHO and United Nation’s family planning guidelines recommending a move towards a more sustainable contraceptive method.^1,18,25^ Woman’s previous exposure to reproductive health services was shown to be a strong determinant of contraceptive use.^26,27,28^ Moreover, Bloom et al. (1999) have found that women who received their antenatal care from professional healthcare workers at healthcare facilities were more likely to use contraceptives in the postpartum period than their counterparts.^28^

Another potential predictor was the woman’s number of living children (*p* = 0.05). The number of living children has been associated with the choice of contraception in several studies.^20,21,29^

A striking finding emerged from the present study is that the outcomes could go either ways.

The non-significance alternative shift suggested by the regression analysis could be consistent with the imbalance between supply and demand factors secondary to the ongoing contraceptive crisis or shortage across South Africa during the data collection period.^30^ These findings suggested that women attending public healthcare facilities (clinics and hospitals) during the crisis period that started in the beginning of 2018 were offered a contraceptive method that was readily available at the clinic or hospital, rather than the one that matched their individual contraceptive demand profile.

A similar mismatch tendency was also observed in Mexico, Brazil and in low- and middle-income countries.^31,32,33^ In these settings, the observed mismatch between the offer and the demand suggested a deficiency in access to a full range of contraceptive methods or a persistent tendency of people to rely on available methods, even though the initial factors underlying the use of methods were no longer relevant.

These observations could equally apply to the women in Odi District area during the study period and subsequently compelling a move towards a reengineering of family planning programmes.

Another striking finding emerged from the observation is that ‘condom’ usage was significantly associated with other family planning methods and was used across all other methods (up to 72.3% of users). The shift towards dual protection method observed in the present study amongst respondents is a crucial step towards the achievement of maternal and reproductive sustainable developmental goals in view of the higher burden of HIV and AIDS in South Africa.^34,35^

## Study limitations

This was a cross-sectional study and might not reflect causal relationships. Another limitation could be the fact that it was a hospital-based population study as women who did not seek medical attention at Odi District Hospital during the survey period were excluded.

## Conclusion

Family planning is an ever evolving service and data on contraception use could assist in strengthening and expanding service delivery. This study revealed a discrepancy within the maternal health delivery system regarding supply and demand chain. The source of family planning information and previous exposure to contraceptive methods were the most influential factors, although the number of living children was a variable determinant.

Therefore, the following policy implications could be drawn from the findings of this study:

- Promoting rational use of contraceptive methods should always be facilitated by qualified healthcare workers at every contact.
- In an effort to improving contraceptives uptake and reducing the burden related to unwanted pregnancies, these findings suggest an evidence-based reengineering of maternal health delivery system.
- The supply chain of contraceptives must tally with the needs identified within the community.
